# Model based on preoperative clinical characteristics to predict lymph node metastasis in patients with gastric cancer

**DOI:** 10.3389/fsurg.2022.976743

**Published:** 2022-09-23

**Authors:** Baicheng Ding, Panquan Luo, Jiahui Yong

**Affiliations:** ^1^ Department of Emergency Surgery, The First Affiliated Hospital of Anhui Medical University, Hefei, China; ^2^ Department of General Surgery, The First Affiliated Hospital of Anhui Medical University, Hefei, China; ^3^ Department of Transfusion, The First Affiliated Hospital of USTC, Division of Life Sciences and Medicine, University of Science and Technology of China, Hefei, China

**Keywords:** lymph node metastasis, gastric cancer, nomogram, preoperative prediction, clinical characteristics

## Abstract

**Background:**

The risk factors of lymph node metastasis (LNM) in gastric cancer (GC) remain controversial. We aimed to identify risk factors of LNM in GC and construct a predictive model.

**Methods:**

A total of 1,337 resectable GC patients who underwent radical D2 lymphadenectomy at the first affiliated Hospital of Anhui Medical University from January 2011 to January 2014 were retrospectively analyzed and randomly divided into training and validation cohorts (*n* = 1,003 and *n* = 334, respectively) in a 3:1 ratio. Collecting indicators include age, gender, body mass index (BMI), tumor location, pathology, histological grade, tumor size, preoperative neutrophils to lymphocytes ratio (NLR), platelets to lymphocytes ratio (PLR), fibrinogen to albumin ratio (FAR), carcinoembryonic antigen (CEA), cancer antigen19-9 (CA19-9) and lymph nodes status. Significant risk factors were identified through univariate and multivariate logistic regression analysis, which were then included and presented as a nomogram. The performance of the model was assessed with receiver operating characteristic curves (ROC curves), calibration plots, and Decision curve analysis (DCA), and the risk groups were divided into low-and high-risk groups according to the cutoff value which was determined by the ROC curve.

**Results:**

BMI, histological grade, tumor size, CEA, and CA19-9 were enrolled in the model as independent risk factors of LNM. The model showed good resolution, with a C-index of 0.716 and 0.727 in the training and validation cohort, respectively, and good calibration. The cutoff value for predicted probability is 0.594, the proportion of patients with LNM in the high-risk group was significantly higher than that in the low-risk group. Decision curve analysis also indicated that the model had a good positive net gain.

**Conclusions:**

The nomogram-based prediction model developed in this study is stable with good resolution, reliability, and net gain. It can be used by clinicians to assess preoperative lymph node metastasis and risk stratification to develop individualized treatment plans.

## Background

Gastric cancer (GC) had the fifth-highest incidence rate and was the third leading cause of cancer-related deaths worldwide (1), Lymph node metastasis (LNM) is the most common way of metastasis in gastric cancer and is one of the important factors affecting the prognosis of patients with GC (2, 3). The Japanese Gastric Cancer Association (JGCA) and the National Comprehensive Cancer Network (NCCN) guidelines have different treatment strategies for the occurrence of LNM in GC (4, 5). The diagnosis of LNM helps formulate surgical plans to determine the extent of lymph node dissection and the need for preoperative chemoradiotherapy (6). However, preoperative diagnosis of LNM is still difficult. LNM in GC mostly occurs in small-sized lymph nodes, and imaging methods such as computed tomography have low specificity (7). The final diagnosis requires pathology.

Some previous studies (8–10) have constructed predictive models for lymph node metastasis in gastric cancer, but they include postoperative data such as tumor invasion depth, lymphovascular invasion, and detailed gastroscopy reports. In some areas with relatively backward inspection levels, complete data may not be available. Therefore, we are committed to screening the risk factors of LNM in GC from simple and readily available preoperative data and building a predictive model for clinical reference.

## Materials and methods

This research was approved by the Ethical Committee and Institutional Review Board of the First Affiliated Hospital of Anhui Medical University and was conducted following the ethical standards of the Helsinki Declaration. Patients were analyzed retrospectively during the research.

### Data source

Clinical data of 1,337 patients with resectable GC who underwent radical D2 lymphadenectomy at the First Affiliated Hospital of Anhui Medical University between January 2011 and January 2014 were retrospectively collected. The inclusion criteria are as follows: (1) resectable gastric cancer; (2) received radical gastrectomy combined with D2 lymph node dissection; (3) preoperative examination including gastroscopy and CT; (4) pathology of gastroscopy including histological morphology and grade; (5) peripheral blood test within 1 week before surgery. The exclusion criteria included; (1) combined with other tumors; (2) radiotherapy or chemotherapy before surgery; (3) the patient has certain diseases that may affect peripheral blood cell counts, such as infections, and blood diseases; (4) number of examined lymph nodes (ELNs) was insufficient (<15); (5) incomplete data.

### Data collection

Data on demographic and clinicopathological characteristics of patients were collected through the medical records office of our hospital, including age, gender, body mass index (BMI), tumor location and size reported by computed tomography (CT), pathology after gastroscopy, peripheral blood test results and postoperative pathology. The tumor size is the largest diameter of the tumor recorded by CT, and the tumor location is determined by gastroscope, the peripheral blood examination data were as follows, including neutrophils, lymphocytes, platelets, albumin, fibrinogen, carcinoembryonic antigen (CEA), and cancer antigen19-9 (CA19-9). NLR is the strict neutrophil count divided by the strict lymphocyte count, PLR is the strict platelet count divided by the strict lymphocyte count, and FAR is the fibrinogen divided by the albumin count.

### Statistical analysis

A two-sided *P* < 0.05 was considered statistically significant. All data were analyzed using SPSS 23.0 for Windows and R (version 4.1.0).variables are presented by mean ± standard deviation, median (interquartile range), or number (percent). Patients were randomly divided into training and validation cohorts (*n* = 1,003 and *n* = 334, respectively) in a 3:1 ratio to construct and validate the nomogram. Student's *t*-test or the non-parametric Mann–Whitney *U*-test and the chi-square test were applied to compare the variables between the groups. The meaningful risk factors which were selected from univariate analysis were further analyzed by the multivariate logistic regression analysis to confirm independent risk factors and selected for the final model. The ROC analysis was used to compare the predictive ability of the predictive model and meaningful risk factors. The cutoff value of CEA, CA19-9, and predicted probability was determined by the ROC curve in the training cohort, CEA, CA19-9 were transformed into categorical variables, and risk groups were divided into low risk and high risk groups according to the cutoff value. The nomogram was constructed to present the model by using the “rms” R package based on the training cohort, the discrimination which represented the predictive accuracy of the nomogram was assessed by the index of concordance (C-index) and internal and externally validated calibration curves, the Hosmer–Lemeshow test was used to evaluate the goodness of fit of the nomogram. Decision curve analysis (DCA) was conducted to determine the clinical usefulness of the nomogram by quantifying the net beneﬁts at different threshold probabilities.

## Results

### Baseline characteristics

The baseline and clinical characteristics analysis of the 1,337 patients are expressed in Table 1. Patients were randomly divided into training and validation cohorts (*n* = 1,003 and *n* = 334, respectively) in a 3:1 ratio. LNM rate in the training cohort was 61.5%, and it was 59.9% in the validation cohort, there were no significant differences in clinical characteristics between the training and validation cohorts.

**Table 1:** Baseline and clinical characteristics of patients in training cohort and validation cohort.

Characteristics	Training set			Validation set			*P* value
	Overall (*n* = 1,003)	LNM (+) (*n* = 617)	LNM (−) (*n* = 386)	Overall (*n* = 334)	LNM (+) (*n* = 200)	LNM (−) (*n* = 134)	
Age (years) (mean ± SD)	60.5 ± 10.7	60.4 ± 10.6	60.5 ± 10.8	61.6 ± 10.2	60.4 ± 10.2	63.5 ± 10.0	0.142
Gender (*n*) (%)							0.549
Male	630 (62.8)	380 (61.6)	250 (64.8)	203 (60.8)	115 (57.5)	88 (65.7)	
Female	373 (37.2)	237 (38.4)	136 (35.2)	131 (39.2)	85 (42.5)	46 (34.3)	
BMI (kg/m^2^) (*n*) (%)							0.920
<18.5	109 (10.9)	83 (13.5)	26 (6.7)	39 (11.7)	26 (13.0)	13 (9.7)	
≥18.5 and <24	652 (65)	395 (64.0)	257 (66.6)	215 (64.4)	138 (69.0)	77 (57.5)	
≥24	242 (24.1)	139 (22.5)	103 (26.7)	80 (24)	36 (18.0)	44 (32.8)	
Tumor location (*n*) (%)							0.361
Upper	546 (54.4)	344 (55.8)	202 (52.3)	173 (51.8)	99 (49.5)	74 (55.2)	
Middle	156 (15.6)	104 (16.9)	52 (13.5)	63 (18.9)	47 (23.5)	16 (11.9)	
Lower	301 (30.0)	169 (27.4)	132 (34.2)	98 (29.3)	54 (27.0)	44 (32.8)	
Pathology (*n*) (%)							0.430
Adenocarcinoma	958 (95.5)	587 (95.1)	371 (96.1)	325 (97.3)	196 (98.0)	129 (96.3)	
Signet ring cell carcinoma	30 (3.0)	18 (2.9)	12 (3.1)	6 (1.8)	2 (1.0)	4 (3.0)	
Mucinous adenocarcinoma	15 (1.5)	12 (1.9)	3 (0.8)	3 (0.9)	2 (1.0)	1 (0.7)	
Histological grade (*n*) (%)							0.841
Poorly	673 (67.1)	455 (73.7)	218 (56.5)	219 (65.6)	150 (75.0)	69 (51.5)	
Moderate	268 (26.7)	146 (23.7)	122 (31.6)	92 (27.5)	47 (23.5)	45 (33.6)	
Well	62 (6.2)	16 (2.6)	46 (11.9)	23 (6.9)	3 (1.5)	20 (14.9)	
Tumor size (cm) (mean ± SD)	5.0 (3.0, 6.5)	5.0 (3.5, 7.0)	3.5 (2.5, 5.0)	5.0 (3.0, 7.0)	5.5 (4.0, 7.0)	3.5 (2.5, 5.1)	0.323
NLR [Median (25%–75%)]	2.10 (1.57, 2.97)	2.20 (1.65, 3.12)	1.98 (1.50, 2.75)	2.05 (1.60, 2.85)	2.25 (1.67, 3.12)	1.90 (1.52, 2.57)	0.668
PLR [Median (25%–75%)]	130.27 (93.57, 182.35)	133.33 (98.79, 190.02)	123.93 (88.78, 166.08)	125.40 (91.12, 169.48)	130.21 (94.76, 180.15)	118.20 (82.26, 157.43)	0.217
FAR [Median (25%–75%)]	0.08 (0.06, 0.11)	0.08 (0.06, 0.12)	0.08 (0.06, 0.10)	0.08 (0.06, 0.11)	0.08 (0.06, 0.11)	0.08 (0.06, 0.10)	0.715
CEA (ng/ml) (*n*) (%)							0.249
<5.61	741 (73.9)	433 (70.2)	308 (79.8)	258 (77.2)	151 (75.5)	107 (79.9)	
≥5.61	262 (26.1)	184 (29.8)	78 (20.2)	76 (22.8)	49 (24.5)	27 (20.1)	
CA19-9 (KU/L) (*n*) (%)							0.317
<13.15	574 (57.2)	318 (51.5)	256 (66.3)	180 (53.9)	106 (53.0)	74 (55.2)	
≥13.15	429 (42.8)	299 (48.5)	130 (33.7)	154 (46.1)	94 (47.0)	60 (44.8)	

Abbreviation: BMI, body mass index; NLR, neutrophils to lymphocytes ratio; PLR, platelets to lymphocytes ratio; FAR, fibrinogen to albumin ratio; CEA, carcinoembryonic antigen; CA19-9, cancer antigen19-9.

### Risk factors of LNM in GC patients

The univariate and multivariable logistic regression analyses are summarized in Table 2. Based on univariate Logistic regression analysis, the risk factors that may affect lymph node metastasis of gastric cancer with *P* < 0.05 were selected to perform multivariate logistic regression analysis in the training cohort. Subsequently, eight variables, which included BMI, histological grade, tumor size, NLR, PLR, FAR, CEA, and CA19-9 were used to perform a multivariate logistic regression analysis. The multivariate analysis demonstrated that BMI < 18.5 (OR = 2.100, CI = 1.216–3.626), poorly differentiated (OR = 4.885, CI = 2.627–9.085), larger tumor size (OR = 1.240, CI = 1.169–1.316), higher CEA (OR = 1.447, CI = 1.046–2.002), higher CA19-9 (OR = 1.529, CI = 1.151–2.029) were associated with a higher risk of LNM. These relationships were similar in the validation set. Finally, five risk factors aforementioned were included in the final model. The predicted value of LNM was expressed by the following equation:

**Table 2:** Univariate and multivariate analysis of risk factors of LNM in GC.

Factors	Univariate analysis			Multivariate analysis		
	*β*	OR (95% CI)	*P* value	*β*	OR (95% CI)	*P* value
Intercept	-			−2.278		
Age	−0.001	0.999 (0.987, 1.011)	0.829			
Gender	−0.137	0.872 (0.670, 1.136)	0.311			
BMI			0.003*			0.015*
<18.5	0.861	2.366 (1.422, 3.935)	0.001*	0.742	2.100 (1.216, 3.626)	0.008*
≥18.5 and <24	0.130	1.139 (0.844, 1.536)	0.394	0.016	1.016 (0.735, 1.405)	0.923
≥24	Ref.			Ref.		
Tumor location			0.052			
Upper	0.285	1.330 (0.999, 1.771)	0.051			
Middle	0.446	1.562 (1.044, 2.338)	0.030*			
Lower	Ref.					
Pathology			0.355			
Adenocarcinoma	−0.927	0.396 (0.111, 1.411)	0.153			
Signet ring cell carcinoma	−0.981	0.375 (0.087, 1.616)	0.188			
Mucinous adenocarcinoma	Ref.					
Histological grade			<0.001*			<0.001*
Poorly	1.792	6.001 (3.322, 10.839)	<0.001*	1.586	4.885 (2.627, 9.085)	<0.001*
Moderate	1.236	3.441 (1.855, 6.380)	<0.001*	1.144	3.141 (1.639, 6.019)	0.001*
Well	Ref.			Ref.		
Tumor size	0.248	1.281 (1.210, 1.358)	<0.001*	0.215	1.240 (1.169, 1.316)	<0.001*
NLR	0.077	1.080 (1.002, 1.164)	0.044*			
PLR	0.003	1.003 (1.001, 1.005)	0.001*			
FAR	2.160	8.672 (1.634, 46.025)	0.011*			
CEA	0.518	1.678 (1.240, 2.271)	0.001*	0.369	1.447 (1.048, 2.002)	0.027*
CA19-9	0.616	1.852 (1.423, 2.410)	<0.001*	0.424	1.529 (1.151, 2.029)	0.003*

In multivariable analysis, BMI, histological grade, tumor size, CEA, CA19-9 were adjusted in the multivariable analyses. Abbreviation: Ref, reference; BMI, body mass index; NLR, neutrophils to lymphocytes ratio; PLR, platelets to lymphocytes ratio; FAR, fibrinogen to albumin ratio; CEA, carcinoembryonic antigen; CA19-9, cancer antigen19-9.

*means statistically significant.


n(p/1−p)=−2.278+0.742∗(BMI<18.5)+0.016∗(BMI≥18.5and<24)+1.586∗(grade=poorly)+1.144∗(grade=moderate)+0.215∗tumorsize+0.369∗(CEA>5.61)+0.424∗(CA19−9>13.15)

### Validation of the risk factors to predict LNM

**Figure 1:**
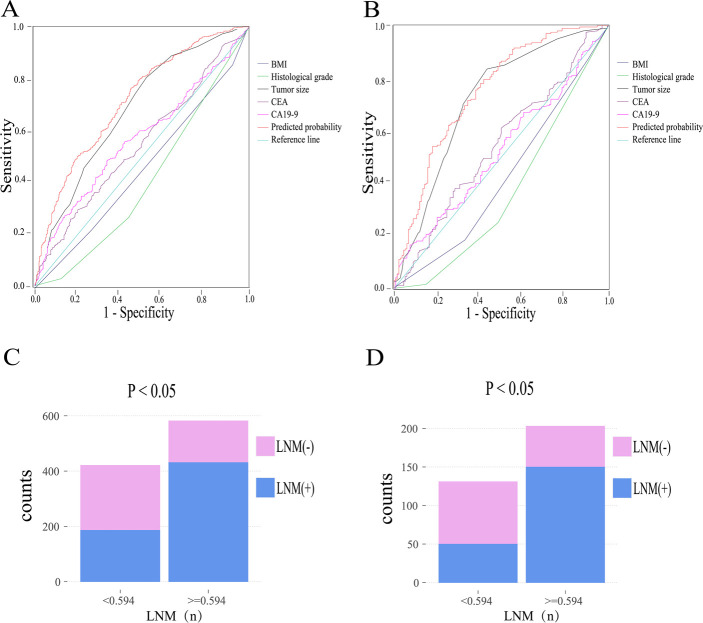
ROC curves and proportion of LNM in the low-risk group and high-risk group in the training cohort (**A,C**). ROC curves and proportion of LNM in the low-risk group and high-risk group in the validation cohort (**B,D**). Abbreviation: BMI, body mass index; CEA, carcinoembryonic antigen; CA19-9, cancer antigen19-9; LNM, lymph node metastasis.

The predictive probability of LNM for each patient in the training and validation cohort was calculated. ROC curves were applied to determine the sensitivity and specificity of the risk factors and the model. As depicted in Figure 1A, The results show that the model demonstrated good resolution with an area under the curve (AUC) of 0.716 (95% CI: 0.683–0.748) in the training cohort, the AUC value of tumor size was 0.682 (95% CI: 0.647–0.716) which was significantly higher than other risk factors (BMI, 0.456; histological grade, 0.404; CEA, 0.549; CA19-9, 0.568). According to the ROC curve evaluation, the optimal cut-off values for the predictive probability of LNM, CEA, and CA19-9 were 0.594, 5.61 ng/ml, and 13.15 U/ml, respectively. According to the cutoff value, the training cohort was divided into low-risk (*n* = 421) and high-risk (*n* = 582) groups. The proportion of patients with LNM in the low-risk group was significantly lower than the high-risk group (*P* < 0.001, Figure 1C). To verify the predictive ability of the final model, ROC curves were also plotted to calculate AUC values for the validation cohort (Figure 1B). The AUC value for the final model in the validation cohort was 0.727 (95% CI: 0.671–0.784). And the AUC values for the tumor size were higher than other factors in the validation cohort (tumor size, 0.718; BMI, 0.422; histological grade, 0.367; CEA, 0.542; CA19-9, 0.522) similarly. the validation cohort was divided into low- (*n* = 131) and high-risk (*n* = 203) groups based on the same cutoff value. The proportion of LNM patients in the high-risk group was also significantly higher than that in the low-risk group (*P* < 0.001, Figure 1D).

### The relationship between tumor size and LNM

**Figure 2:**
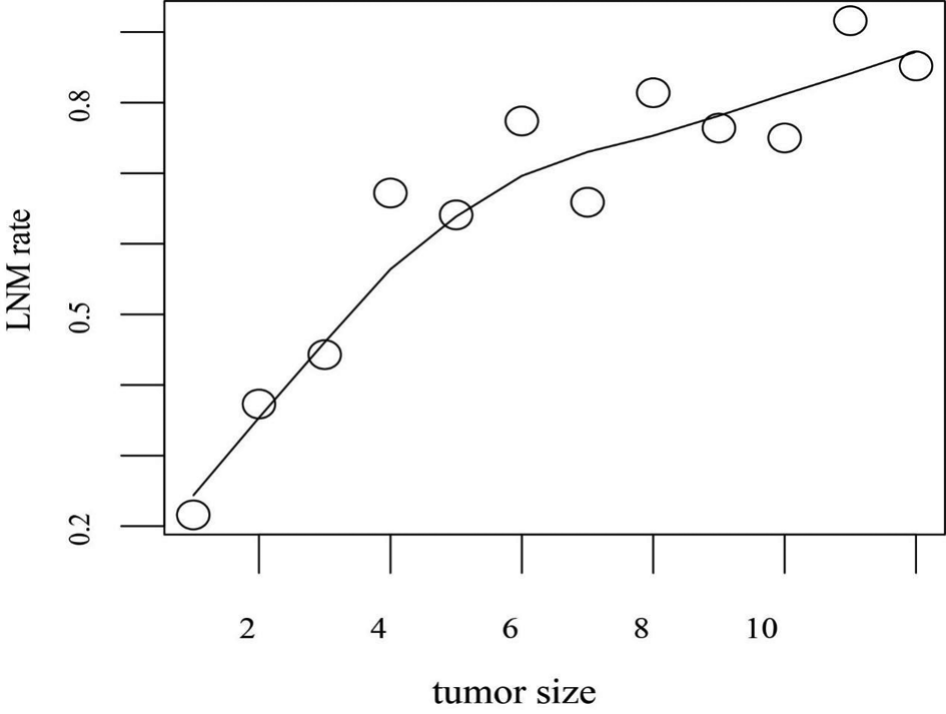
Tumor size counts are recorded over 1-cm intervals. LNM rate refers to the proportion of patients with LNM to all patients in the training set within the range of unit tumor size. LNM rates are adjusted for tumor size. Abbreviation: LNM, lymph node metastasis.

To evaluate the influence of tumor size on LNM of GC, the trend graph between tumor size and LNM rate was plotted. With the increase of the tumor size, the lymph node metastasis rates gradually increased (Figure 2). Especially patients whose tumor size was in the range of 10–11 cm have a higher incidence of LNM (91.6%).

### Construction and validation of nomogram

**Figure 3:**
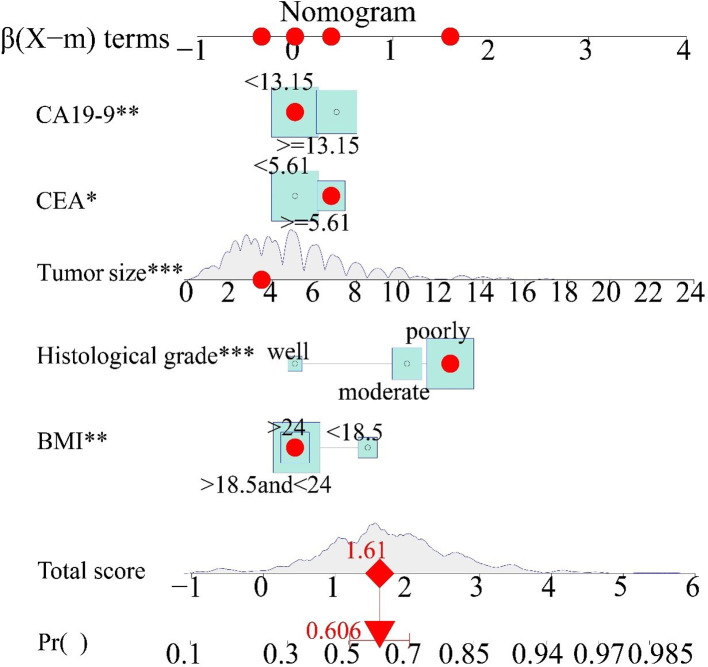
Clinical interactive application. The red points on the five axes of BMI, histological grade, tumor size, CEA, and CA19-9 represent individual patient independent variable scores, which were selected by the results of the preoperative examination. The red dot on the total score represents the total score of the individual patient's LNM risk, and the downward red arrow represents the probability of a specific LNM for that score. BMI, body mass index; CEA, carcinoembryonic antigen; CA19-9, cancer antigen19-9.

A nomogram was used to present the model and facilitate its clinical application by using the interactive package “regplot” based on the training cohort. Medical staff can directly click on any position of the independent variable, and the total score of LNM and predicted probability will be displayed directly, which is very user-friendly. For example, a patient with BMI > 24, poorly histological grade, tumor size of 3.5 cm, CEA ≥ 5.61, CA19-9 < 13.15, the total LNM score is −0.714 and the LNM probability of 60.6% would show immediately (Figure 3).

### Validation of the nomogram

**Figure 4:**
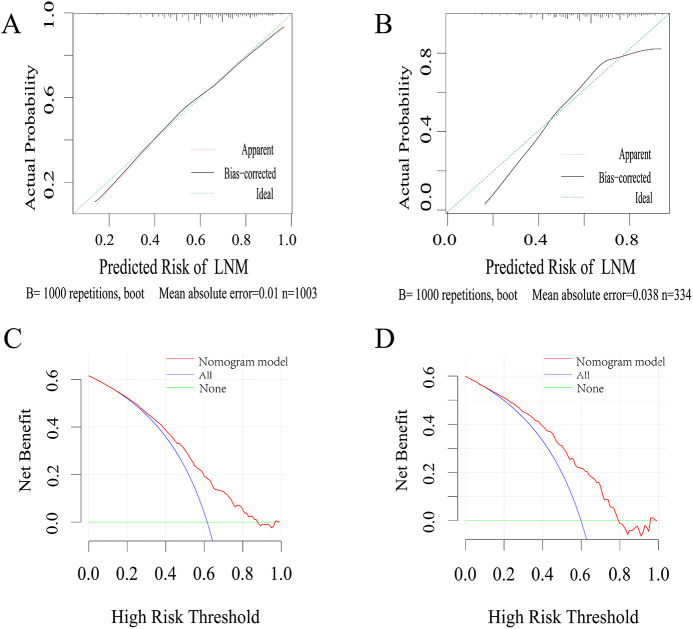
(**A,B**) Calibration curves of the developed nomogram in training and validation cohort. The *x*-axis represents the predicted probability from the nomogram, and the *y*-axis is the actual probability of LNM in GC patients. (**C,D**) DCA of the risk assessment model in the training and validation cohort. The *y*-axis measures the net beneﬁt. The red line represents the predicted nomogram model. The blue line represents the assumption that all GC patients had LMN. The green line represents the assumption that no patients had LMN. The net beneﬁt was calculated by subtracting the proportion of all patients who are false positive from the proportion who are truly positive. LNM, lymph node metastasis.

Calibration curves were used to verify the performance of the model in predicting the risk of LNM. The calibration curve was close to the ideal curve, Hosmer–Lemeshow test *χ*^2^ = 6.814, *P* = 0.557 > 0.05, which indicated that the constructed nomogram has a good degree of calibration (Figures 4A,B). To further validate the performance of the model in clinical applicability, The DCA showed that the nomogram adds more benefit than either the treat-all-patients scheme or the treat-none scheme in predicting LNM when the threshold probability of a patient or doctor ranges between 30% and 85% in the training cohort (while ranges between 20% and 80% in the validation cohort) (Figures 4C,D). Our nomogram demonstrated the net benefit of nomogram-assisted decisions at a wide range of threshold probability.

## Discussion

Radical surgery is the standard treatment for GC (11, 12). Patients with or without LNM have a significantly different prognosis and may require different degrees of lymph node dissection or neoadjuvant therapy. Accurate diagnosis of LNM is one of the foundations of individualized treatment of GC, and it is also of great significance for formulating surgical strategies and assessing the prognosis of GC patients. European Society of Medical Oncology (ESMO) and National Comprehensive Cancer Network (NCCN) guidelines recommend the use of medical imaging for preoperative N staging (5, 13). In particular, multidetector computed tomography (MDCT) imaging has been routinely used for preoperative N staging, lymphadenopathy and roundness were signs of LNM. However, the accuracy of MDCT for LNM was about 50%–70% (14), which is unsatisfactory. Therefore, we filtered out LNM independent predictors by using easy-to-obtain preoperative indicators. Finally, we incorporated the clinical risk factors including BMI, histological grade, tumor size, CEA, and CA19-9 into a nomogram to establish a risk assessment model for LNM in GC. The model showed good resolution, with a C-index of 0.716 and 0.727 in the training and validation cohort, respectively, and good calibration. DCA showed that the clinical utilities of this model were excellent.

ROC curves were also applied to determine the sensitivity and specificity of the risk factors, the AUC value of tumor size was significantly higher than other risk factors. The trend graph showed a clear positive correlation between tumor size and risk of lymph node metastasis, larger tumors would have a higher risk of LNM. Habermann et al. (15) proposed that tumor size, depth of invasion, and degree of differentiation were important clinicopathological factors which should be considered discreetly in the risk of LNM. Chao Huang et al. (10) retrospectively analyzed the clinical data of 554 GC patients who underwent gastrectomy with D2 lymphadenectomy and found that tumor size, CT findings, histological grade, Hb, CEA, and CA19-9 were independent risk factors of LNM. and tumor size was found to be the most important factor for the evaluation of LNM through a random forest algorithm and classification tree which consistent with the conclusion of our research.

In GC, The role of BMI in LNM seems to be controversial so far. Studies have shown that high BMI increases the risk of EGC or mucosal dysplasia, in which insulin resistance plays an important role (16, 17). However, other studies have shown that BMI was inversely associated with tumor size, tumor depth, LNM, tumor stage, and prognosis in early or advanced GC (18, 19). To explore whether the above controversy is related to the grouping of patients, Yi Zou et al. (20) evaluated the role of BMI in underweight (<18.5 kg/m^2^), normal (18.5–24 kg/m^2^), or overweight (≥24 kg/m^2^), and found that all three groups had sustained protective effects. We could not make such a conclusion, unfortunately. Our results indicated that BMI is a protective factor for LNM in GC, once a tumor occurs, it may consume excessive energy, and sufficient energy storage might be essential for the anti-tumor response.

The preoperatively elevated serum CEA and CA19-9 levels were significantly associated with LNM in GC and could be used as reliable biomarkers for predicting LNM in GC (21, 22). In terms of clinical features, elevated levels of CEA and CA19-9 indicated increased tumor invasiveness and metastasis (23). Keshen Wang et al. (21) indicated that CEA has important value in the diagnosis of LNM in GC. In our study, tumor markers were also correlated with LNM. we found that preoperative CEA level and CA19-9 level were independent risk factors for LNM in GC. NLR and PLR are common serum markers, they can reflect the state of inflammation, and they are related to the prognosis of GC patients (24). Few studies were comparing the risk factor of LNM with NLR (PLR). Our univariate analysis showed that both NLR and PLR are risk factors for LNM, but to our surprise, they were not independent risk factors.

In this study, we developed a risk assessment model for LNM in GC. The results showed that the model could effectively predict the incidence of LNM in GC and had good clinical application capabilities. The novel nomogram was useful to assess the LNM of patients that have undergone gastrectomy and to help determine the appropriate treatment. For example, high-risk scores of patients should have more attention to the treatment strategies after surgery.

This study has several limitations. First, it is a retrospective study, the inevitable selection bias should be addressed in future prospective and externally validated studies. Secondly, the established nomogram model has clinical value in the diagnosis of LNM in GC, but its ability to accurately predict the number of LNM is limited.

## Conclusion

In summary, BMI, histological grade, tumor size, CEA, and CA19-9 were independent risk factors for LNM in GC. The new nomogram model developed based on these factors provided a very useful predictive tool for individual prediction of LNM of GC patients.

## Data Availability

The original contributions presented in the study are included in the article/Supplementary Material, further inquiries can be directed to the corresponding author/s.
